# Piscidin is Highly Active against Carbapenem-Resistant *Acinetobacter baumannii* and NDM-1-Producing *Klebsiella pneumonia* in a Systemic Septicaemia Infection Mouse Model

**DOI:** 10.3390/md13042287

**Published:** 2015-04-14

**Authors:** Chieh-Yu Pan, Jian-Chyi Chen, Te-Li Chen, Jen-Leih Wu, Cho-Fat Hui, Jyh-Yih Chen

**Affiliations:** 1 Department and Graduate Institute of Aquaculture, National Kaohsiung Marine University, Kaohsiung, Taiwan 811, Taiwan; E-Mail: panjade@webmail.nkmu.edu.tw; 2 Department of Biotechnology, Southern Taiwan University, 1 Nan-Tai Street, Yung-Kang City, Tainan County 710, Taiwan; E-Mail: jianchyi@mail.stut.edu.tw; 3 School of Medicine, National Yang-Ming University, No.155, Sec.2, Linong Street, Taipei 112, Taiwan; 4 Institute of Cellular and Organismic Biology, Academia Sinica, Taipei 112, Taiwan; E-Mails: jlwu@gate.sinica.edu.tw (J.-L.W.); scfhui@gate.sinica.edu.tw (C.-F.H.); 5 Marine Research Station, Institute of Cellular and Organismic Biology, Academia Sinica, 23-10 Dahuen Road, Jiaushi, Ilan 262, Taiwan

**Keywords:** antimicrobial peptide, piscidin, *Acinetobacter baumannii*, *Klebsiella pneumonia*

## Abstract

This study was designed to investigate the antimicrobial activity of two synthetic antimicrobial peptides from an aquatic organism, tilapia piscidin 3 (TP3) and tilapia piscidin 4 (TP4), *in vitro* and in a murine sepsis model, as compared with ampicillin, tigecycline, and imipenem. Mice were infected with (NDM-1)-producing *K. pneumonia* and multi-drug resistant *Acinetobacter baumannii*, and subsequently treated with TP3, TP4, or antibiotics for different periods of time (up to 168 h). Mouse survival and bacterial colony forming units (CFU) in various organs were measured after each treatment. Toxicity was determined based on observation of behavior and measurement of biochemical parameters. TP3 and TP4 exhibited strong activity against *K. pneumonia* and *A. baumannii in vitro*. Administration of TP3 (150 μg/mouse) or TP4 (50 μg/mouse) 30 min after infection with *K. pneumonia* or *A. baumannii* significantly increased survival in mice. TP4 was more effective than tigecycline at reducing CFU counts in several organs. TP3 and TP4 were shown to be non-toxic, and did not affect mouse behavior. TP3 and TP4 are able at potentiate anti-*Acinetobacter baumannii* or anti-*Klebsiella pneumonia* drug activity, reduce bacterial load, and prevent drug resistance, indicating their potential for use in combating multidrug-resistant bacteria.

## 1. Introduction

Global reports of infections by New Delhi metallo-ß-lactamase 1 (NDM-1)-producing *Klebsiella pneumonia* are increasing; the β-lactamase produced by this strain is particularly worrying, as it is able to inactive all β-lactams, with the exception of aztreonam [1]. NDM-1-producing *Klebsiella pneumonia* was first identified in India in 2008, and has since disseminated at a high rate among Enterobacteriaceae isolates in India and other countries [2,3,4]. These NDM-1-producing strains are a source of community-acquired urinary tract and nosocomial infections, which can result in serious septicaemia and ventilator associated pneumonia (VAP) [1]. At present, drugs capable of treating infections caused by NDM-1-producing *Klebsiella pneumonia* are limited to colistin and tigecycline [2]. Another bacterial species, *Acinetobacter baumannii*, is known for its ability to cause hospital infections and its resistance to most antibiotics [5]. First-line treatment of *Acinetobacter baumannii* infections usually involves administration of imipenem, a carbapenem antibiotic. However, cases of carbapenem-resistant *Acinetobacter baumannii* are increasing. Alternative drugs for treatment include polymyxins, tigecycline, and aminoglycosides [6]. However, a large number of *A. baumannii* strains have also developed resistance to these antibiotics. These multidrug-resistant pathogens are extremely difficult to treat and pose a serious threat to health care; as such, there is a vital need for new effective therapeutics to tackle infections caused by drug-resistant bacteria [7].

The ubiquity of antimicrobial peptides (AMPs) in nature attests to their overall importance in the defense strategies of most organisms. The extraordinary distribution of AMPs in all kingdoms suggests that they have played a fundamental role in the evolution of complex multicellular organisms. They are considered part of the humoral natural defense of invertebrates against infections, and have thus been termed “natural antibiotics” [8]. Invertebrate AMPs interact with certain primitive functions of acquired immunity, such as cytokine signal transduction, TLR interaction, and ancient immune memory, thus acting as effective immunomodulators [9,10]. In addition, recent findings suggest that AMPs are capable of playing multifunctional roles that extend beyond their capacity to act as gene-encoded antibiotics and anticancer agents [11]; certain AMPs have been shown to effectively stimulate the immune system by favoring cytokine release, chemotaxis, antigen presentation, angiogenesis, inflammatory responses, and adaptive immune induction [12]. Much of the research on AMPs over the past decade has focused on bio-prospecting novel compounds with activity against a wide range of microorganisms, including Gram-positive and -negative bacteria, fungi, viruses, protozoa, and parasites (such as nematodes) [13]. Over the past decade, over 20 new or modified antibiotics have been released into the market, and over 40 AMPs are currently under active clinical development [14,15]. Several synthetic AMPs have entered clinical trials, and many peptides or mimetics are undergoing (with a few having completed) clinical trials as antimicrobials or immunomodulatory agents [14,16]. Recently, one family of AMPs, piscidins, have attracted attention as peptides with broad bactericidal activity [17]. The piscidin family includes pleurocidin, moronecidin, chrysophsin, dicentracin, epinecidin-1, and myxinidin. Piscidins have been identified in acidophilic granulocytes, which possess functional equivalence to neutrophils in higher vertebrates [18]. Synthetic piscidins have reported activity against bacteria, fungi, and parasites; for example, piscidins have anti-parasitic activity against trophonts (*C. irritans*), a marine fish ectoparasite. Additional studies have indicated that piscidins may be an important component of the innate immune system against *C. irritans* and secondary bacterial infections [13,17,19].

Recently, we isolated several novel AMPs, including five piscidin cDNA clones from tilapia (*Oreochromis niloticus*) spleen; these clones were named tilapia piscidin 1 (TP1), TP2, TP3, TP4, and TP5 [17]. TP3 and TP4 exhibited potent, broad-spectrum antimicrobial activities, and no noticeable hemolytic activity. These features suggest that TP3 and TP4 may be suitable for development as novel, systemically-administered antimicrobial agents. However, the efficacy of TP3 and TP4 against infections caused by NDM-1-producing Enterobacteriaceae or multidrug-resistant (MDR) *A. baumannii* strains *in vivo* remains unclear. Thus, the aim of this study was to estimate the efficacy of these antimicrobials in an experimental model of sepsis caused by NDM-1-producing *Klebsiella pneumonia* and multidrug-resistant (MDR) *A. baumannii*.

## 2. Results

### 2.1. In Vitro Efficacy of TP3 and TP4

**Figure 1:**
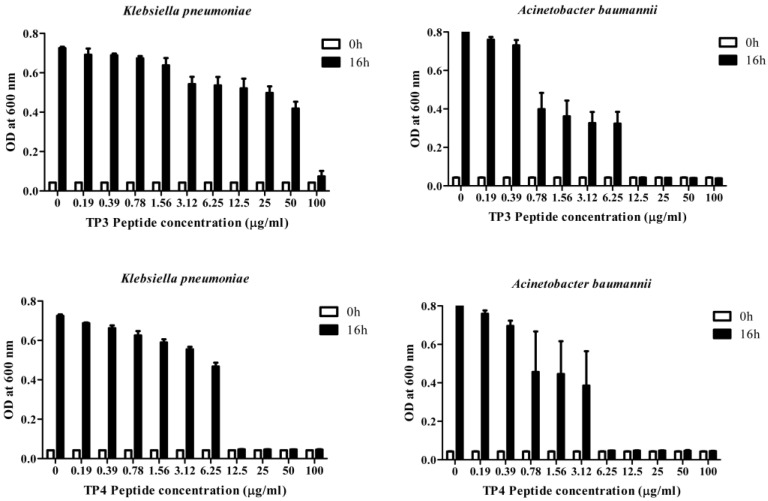
Dose-dependent growth inhibition of clinical isolates of carbapenem-resistant *Acinetobacter baumannii* (Sk44) and NDM-1-producing *Klebsiella pneumonia* (NDM-1) by incubation with TP3 or TP4 for 16 h. The data are expressed as means of three replicates. Error bars represent the standard derivation (SD). OD, optical density.

**Figure 2:**
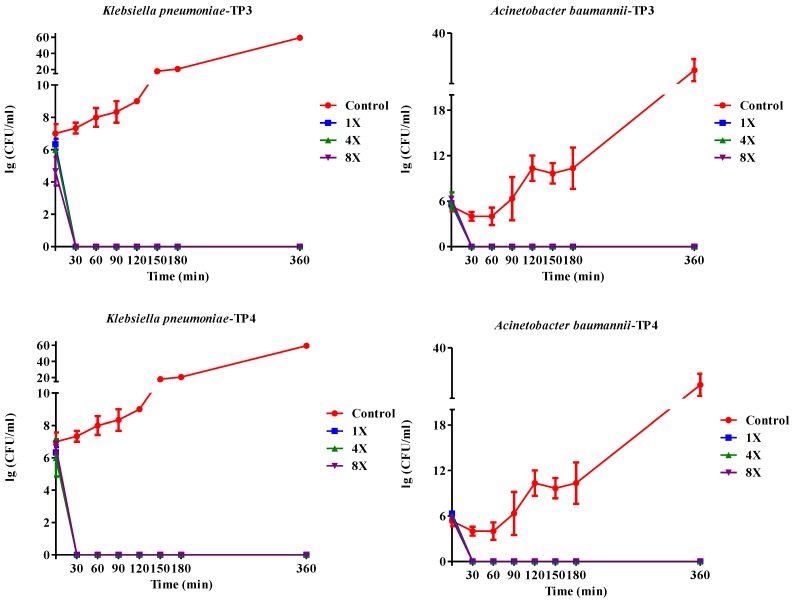
Kill kinetics of TP3 and TP4 against Acinetobacter baumannii (Sk44) and Klebsiella pneumonia (NDM-1). Controls were not treated with peptide. The peptide concentrations were 1 × MIC (1×), 4 × MIC (4×), or 8 × MIC (8×). Experiments were performed in triplicate and the bactericidal activities are presented as mean lg (CFU/mL). Error bars represent the standard deviations.

We first determined the minimum inhibitory concentrations (MICs; Table 1a) and minimum bactericidal concentrations (MBCs; Table 1b) of TP3 or TP4 against clinical isolates of *A. baumannii*, *E. coli*, or *K. pneumonia* (Supplementary Table S1) The *A. baumannii* isolates were more susceptible to TP4 than to TP3, piscidin-1, or imipenem, while the *K. pneumonia* isolates were more susceptible to TP3 and TP4 than to piscidin-1 or imipenem (Table 1). However, TP4 and ampicillin were unable to inhibit growth of the *K. pneumonia* (blaNDM-1) strain. The MBC values of TP3 and TP4 against all tested clinical isolates ranged from 1.56–3.125 μg/mL. *A. baumannii* (Sk44) and *K. pneumonia* (NDM-1) were selected as representative strains for subsequent experiments. We proceeded to identify that TP4 is more effective than TP3 at suppressing the growth of *A. baumannii* (Sk44), *K. pneumonia* (NDM-1), *P. aeruginosa* (19,660), and *P. aeruginosa* (R) (Figure 1; Supplementary Figure S1). We further examined the bactericidal effects of TP3 and TP4 against *A. baumannii* (Sk44), *K. pneumonia* (NDM-1), *P. aeruginosa* (19,660), and *P. aeruginosa* (R) by determining their time-kill curves. TP3 and TP4 exhibited a dose-dependent bactericidal effect, defined as a ≥3 lg decrease in the initial inoculum (Figure 2; Supplementary Figure S2). Interesting, TP4 time-kill curves were similar with those of TP3. All bacterial strains were completely eradicated by TP3 and TP4 by 60 min of exposure at MIC.

**Table 1:** Antimicrobial activity of TP3 and TP4 against clinical isolates of *A. baumannii*, *E. coli*, or *K. pneumonia*, as compared with that of piscidin-1 and commonly used antimicrobial agents. (**a**) Minimum inhibitory concentrations (MIC). (**b**) Minimum bactericidal concentrations (MBC). ND: not determined.

(a)					
Bacterial Strain	TP3	TP4	Piscidin-1	Ampicillin	Imipenem
	(μg/mL)	(μg/mL)	(μg/mL)	(μg/mL)	(μg/mL)
K. pneumoniae (YT32)	3.125	3.125	50	ND	<1.56
E. coli (YT39)	<1.56	<1.56	25	ND	<1.56
E. coli (YT154)	<1.56	<1.56	3.125	ND	<1.56
A. baumannii (Icu53)	<1.56	<1.56	6.25	ND	<1.56
A. baumannii (Sk44)	12.5	<1.56	3.125	ND	50
K. pneumoniae (NDM-1)	25	3.125	50	ND	3.125
K. pneumoniae (blaNDM-1)	3.125	ND	50	ND	<1.56
**(b)**					
**Bacterial Strain**	**TP3**	**TP4**	**Piscidin-1**	**Ampicillin**	**Imipenem**
	**(μg/mL)**	**(μg/mL)**	**(μg/mL)**	**(μg/mL)**	**(μg/mL)**
*K. pneumoniae* (YT32)	3.125	3.125	50	ND	1.56
*E. coli* (YT39)	1.56	1.56	25	ND	1.56
*E. coli* (YT154)	1.56	1.56	3.125	ND	1.56
*A. baumannii* (Icu53)	1.56	1.56	6.25	ND	12
*A. baumannii* (Sk44)	25	1.56	25	ND	50
*K. pneumoniae* (NDM-1)	25	3.125	50	ND	6
*K. pneumoniae* (blaNDM-1)	3.125	ND	50	ND	6

**Figure 3:**
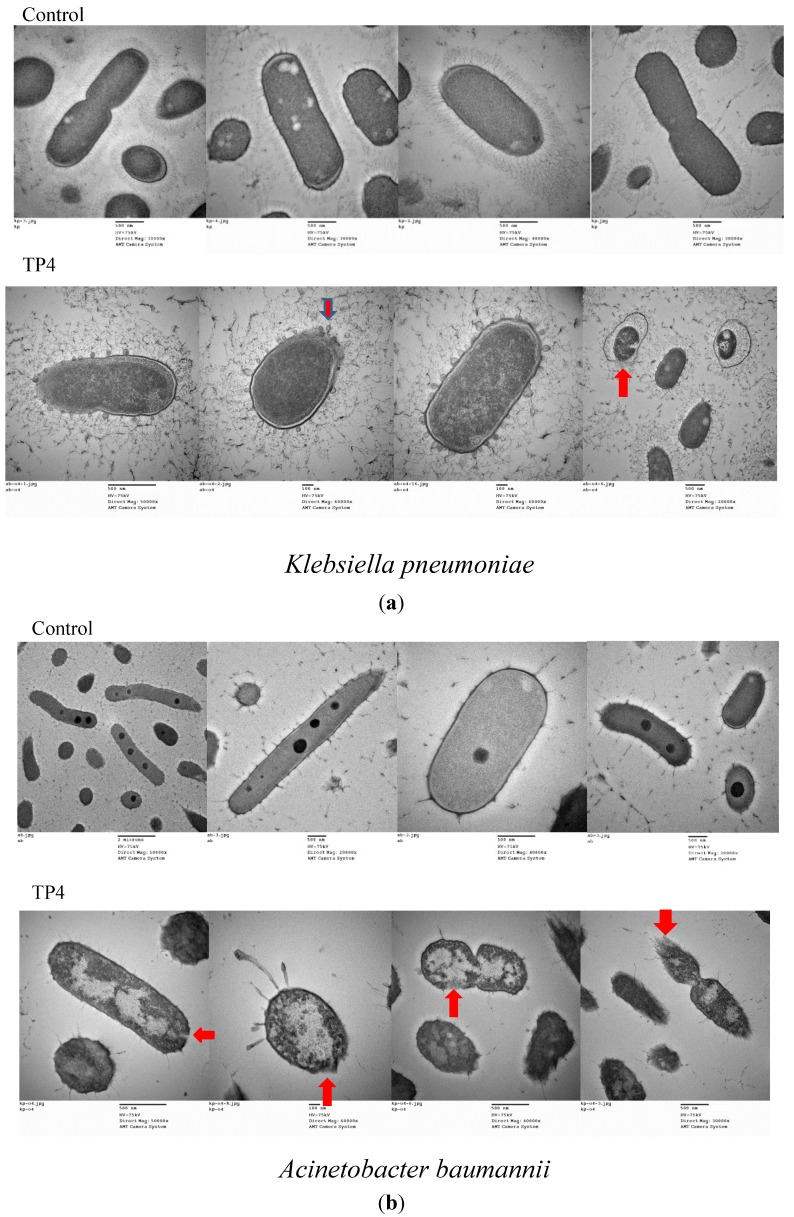

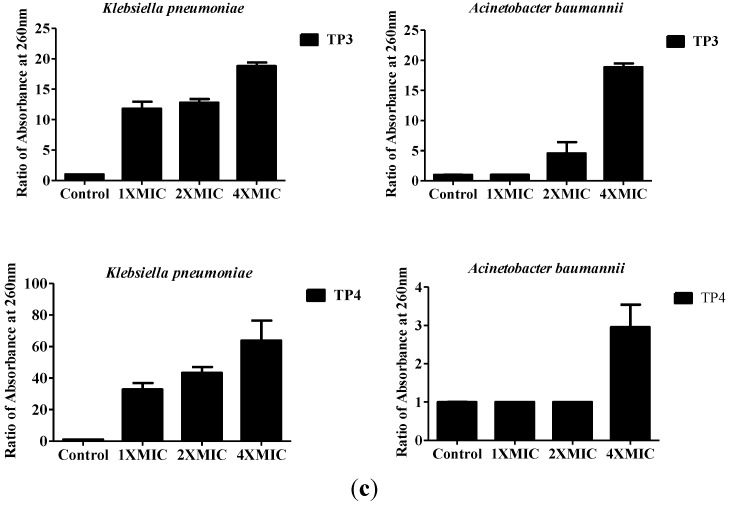
The effects of TP3 and TP4 on bacterial cytoplasmic membrane integrity. (**a**) Transmission electron microscopy (TEM) of Klebsiella pneumonia (NDM-1) incubated in control broth (Control) or broth containing TP4 (100 μg/mL) at 37 °C for 60 min; (**b**) TEM of Acinetobacter baumannii (Sk44) incubated in control broth (Control) or broth containing TP4 (100 μg/mL) at 37 °C for 60 min; (**c**) The effect of treatment with TP3 or TP4 on the release of DNA from the cytoplasm of Acinetobacter baumannii (Sk44) and Klebsiella pneumonia (NDM-1). Cells were treated with peptides at concentrations of 1 × MIC, 2 × MIC, or 4 × MIC. The data shown are the means of at least three independent experiments. Error bars represent the standard deviations.

We next examined the effect of treatment with TP3 or TP4 on membrane composition by TEM (Figure 3a,b) and cellular leakage experiments (Figure 3c). In contrast to the continuous membrane structure of untreated bacteria, the cytoplasmic membranes of TP4-treated cells were disrupted, allowing cellular contents to escape (Figure 3a,b). To confirm that cytoplasmic leakage had occurred, we measured the absorbance of the growth media at 260 nm (indicating the release of nucleic acid from bacteria) before and after peptide treatment. As shown in Figure 3C, TP3 and TP4 exerted a dose-dependent effect on the release of cytoplasmic content from *A. baumannii* (Sk44) and *K. pneumonia* (NDM-1) cells. Similar results were observed using *P. aeruginosa* (R) and *P. aeruginosa* (19,660) (Supplementary Figure S3), suggesting that TP3 and TP4 exert their bactericidal activity in part by inducing membrane lysis.

### 2.2. In Vivo Efficacy of TP3 and TP4 against Infections Caused by A. Baumannii (Sk44) and K. Pneumonia (NDM-1)

To study the bactericidal effects of TP3 and TP4 *in vivo*, we monitored their effect on the survival of mice infected with *A. baumannii* (Sk44) or *K. pneumonia* (NDM-1) (Materials and Methods). At 7 days after *A. baumannii* (Sk44) infection, the survival rate was 0%, 62.2%, 93.3%, 8.8%, 46.6%, and 65.5% for mice treated with PBS, TP3 (150 μg/mouse), TP4 (50 μg/mouse), ampicillin (150 μg/mouse), imipenem (150 μg/mouse), and tigecycline (150 μg/mouse), respectively (Table 2). On the other hand, at 7 days after *K. pneumonia* (NDM-1) infection, the survival rate was 0%, 71.1%, 88.8%, 0%, 45.5%, and 46.6% for mice treated with PBS, TP3 (150 μg/mouse), TP4 (50 μg/mouse), ampicillin (150 μg/mouse), imipenem (150 μg/mouse), and tigecycline (150 μg/mouse), respectively (Table 2).

**Figure 4:**
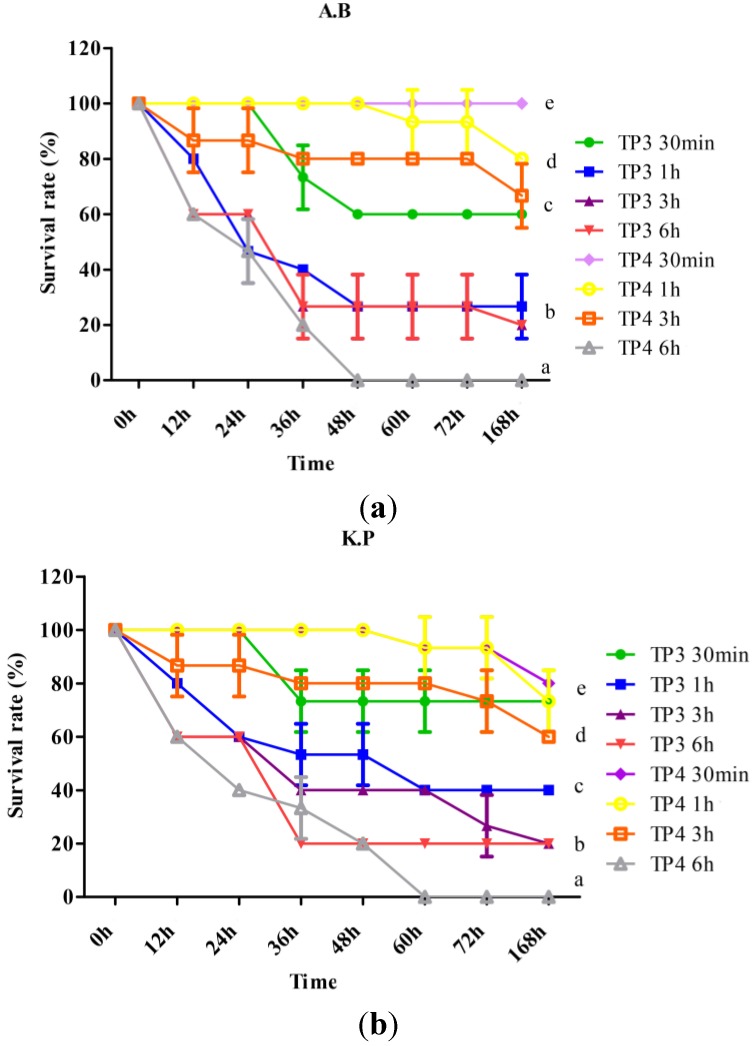
Effects of treatment with TP3 and TP4 on (**a**) Acinetobacter baumannii (Sk44) (A.B) and (**b**) Klebsiella pneumonia (NDM-1) (K.P) infection in mice. Mice were injected with bacteria, and TP3 or TP4 were subsequently injected at the indicated times (30 min, 1 h, 3 h, or 6 h) after infection (*n* = 5 for each group). The survival rate was monitored on a daily basis for up to 168 h. Data represent the means, and error bars represent standard deviations.

To determine curative potential, mice were first injected with *A. baumannii* (Sk44) or *K. pneumonia* (NDM-1), and then injected with TP3 (150 μg/mice) or TP4 (50 μg/mice) at 0.5, 1, 3, or 6 h after infection. We observed that the earlier TP3 or TP4 was added, the higher the survival rate, for mice infected with *A. baumannii* (Sk44) (Figure 4a) or *K. pneumonia* (NDM-1) (Figure 4b). These data indicate that immediate application of TP3 or TP4 are essential to prevent severe infection in mice.

**Table 2:** Survival of mice infected with K. pneumonia (NDM-1) (KP) or A. baumannii (Sk44) (AB) upon treatment with TP3, TP4, ampicillin (AMP), tigecycline (TIG), imipenem (IMP), or PBS (control). Mice were infected with A. baumannii (Sk44) (3 × 107 CFU/mL, 100 μL/mouse) or K. pneumonia (NDM-1) (1.5 × 108 CFU/mL, 100 μL/mouse), and then treated with the indicated compound 30 min later. Survival rate (%) at 168 h after the commencement of treatment is shown.

Treatment	Survival (%) KP	Survival (%) AB
Control	0 ± 0	0 ± 0
TP3	71.1 ± 11.7 (*P* < 0.05)	62.2 ± 3.8 (*P* < 0.05)
TP4	88.8 ± 19.24 (*P* < 0.05)	93.3 ± 11.5 (*P* < 0.05)
AMP	0 ± 0	8.8 ± 8.3
IMP	45.5 ± 32.03 (*P* < 0.05)	46.6 ± 41.6 (*P* < 0.05)
TIG	46.6 ± 41.63 (*P* < 0.05)	65.5 ± 43.5 (*P* < 0.05)

### 2.3. TP3 and TP4 Exhibit in Vivo Bacteriostatic Properties against A. Baumannii (Sk44) and K. Pneumonia (NDM-1)

We next examined the bacteriostatic properties of TP3 and TP4 by examining bacterial load in various mouse organs after infection with *A. baumannii* (Sk44) or *K. pneumonia* (NDM-1) and treatment with the relevant compound. Treatment of *K. pneumonia* (NDM-1) or *A. baumannii* (Sk44) infection with TP4 resulted in a comparable reduction in bacteria in blood to that induced by antibiotics, but a greater reduction in liver samples; TP3 treatment significantly reduced the *A. baumannii* (Sk44) bacterial load in liver as compared to untreated controls or antibiotic-treated mice (Table 3). These data indicate that TP3 or TP4 can efficiently control the multiplication of *A. baumannii* (Sk44) or *K. pneumonia* (NDM-1) in the organs of infected mice.

**Table 3:** Bacterial counts at 72 h after the last treatment in the indicated organs of mice infected with K. pneumonia (NDM-1) (KP) or A. baumannii (Sk44) (AB). Infected mice were untreated, or treated with TP3, TP4, ampicillin, tigecycline (TIG), or imipenem (IMP) via i.p. injection. Bacterial numbers in blood, peritoneum, spleen, liver, and mesenteric lymph nodes were subsequently recorded. Colony counts from the diluted bacterial solutions are expressed relative to those at the start of treatment. Each value represents the mean value from three determinations ± standard derivation (SD). Differences were defined as significant at *p* < 0.05. Different letters indicate a significant difference between two groups, while the same letter indicates no difference between two groups.

Treatment (KP)	Bacterial Count in Blood (CFU/mL)	Bacterial Count in Peritoneum (CFU/mL)	Bacterial Count in Spleen (CFU/mL)	Bacterial Count in Liver (CFU/mL)	Bacterial Count in mesenteric lymph nodes (CFU/mL)
No treatment	4.2 × 10^4^ ± 1.2 × 10^4 c^	3.8 × 10^7^ ± 0.9 × 10^7 e^	6.6 × 10^7^ ± 2.2 × 10^7 e^	6.9 × 10^7^ ± 3.8 × 10^7 e^	6.5 × 10^7^ ± 1.9 × 10^7 e^
TP3 (150 μg/mouse)	2.4 × 10^3^ ± 0.2 × 10^3 b^	1.4 × 10^7^ ± 0.2 × 10^7 e^	1.6 × 10^7^ ± 1.3 × 10^7 e^	1.6 × 10^7^ ± 0.2 × 10^7 e^	2.8 × 10^7^ ± 1.5 × 10^7 e^
TP4 (50 μg/mouse)	0.2 × 10^2^ ± 0.1 × 10^2 a^	8.6 × 10^6^ ± 4.1 × 10^6 d^	2.3 × 10^7^ ± 0.9 × 10^7 e^	1.2 × 10^7^ ± 0.6 × 10 ^7 e^	1.4 × 10^7^ ± 1.1 × 10^7 e^
TIG (100 μg/mouse)	0.2 × 10^2^ ± 0.1 × 10^2 a^	7.0 × 10^6^ ± 6.7 × 10^6 d^	2.3 × 10^7^ ± 1.2 × 10^7 e^	6.2 × 10^6^ ± 1.0 × 10^6 d^	1.5 × 10^7^ ± 0.1 × 10^7 e^
IMP (100 μg/mouse)	2.3 × 10^2^ ± 1.6 × 10^2 a^	9.4 × 10^6^ ± 1.0 × 10^6 d^	1.2 × 10^7^ ± 0.4 × 10^7 e^	4.2 × 10^6^ ± 0.4 × 10^6 d^	1.3 × 10^7^ ± 1.3 × 10^7 e^
AMP (100 μg/mouse)	3.2 × 10^4^ ± 2.0 × 10^4 c^	3.2 × 10^7^ ± 2.4 × 10^7 e^	3.1 × 10^7^ ± 0.5 × 10^7 e^	1.7 × 10^6^ ± 0.4 × 10^7 e^	2.3 × 10^7^ ± 0.5 × 10^7 e^
**Treatment (AB)**	**Bacterial Count in Blood (CFU/mL)**	**Bacterial Count in Peritoneum (CFU/mL)**	**Bacterial Count in Spleen (CFU/mL)**	**Bacterial Count in Liver (CFU/mL)**	**Bacterial Count in mesenteric lymph nodes (CFU/mL)**
No treatment	3.6 × 10^6^ ± 2.3 × 10^6 d^	7.3 × 10^8^ ± 2.3 × 10^8 f^	7.8 × 10^8^ ± 1.2 × 10^8 f^	3.3 × 10^9^ ± 5.9 × 10^8 g^	9.2 × 10^8^ ± 5.4 × 10^8 f^
TP3 (150 μg/mouse)	1.4 × 10^3^ ± 0.1 × 10^3 b^	5.8 × 10^7^ ± 2.9 × 10^7 e^	2.1 × 10^7^ ± 1.0 × 10^7 e^	1.7 × 10^7^ ± 0.8 × 10^7 e^	2.6 × 10^7^ ± 1.0 × 10^7 e^
TP4 (50 μg/mouse)	0.2 × 10^2^ ± 1.0 × 10^2 a^	7.8 × 10^7^ ± 1.1 × 10^7 e^	9.2 × 10^7^ ± 1.4 × 10^7 e^	2.4 × 10^6^ ± 0.4 × 10^6 d^	1.1 × 10^7^ ± 0.1 × 10^7 e^
TIG (100 μg/mouse)	0.4 × 10^2^ ± 0.2 × 10^2 a^	1.4 × 10^7^ ± 0.1 × 10^7 e^	2.3 × 10^7^ ± 0.3 × 10^7 e^	2.8 × 10^7^ ± 0.3 × 10^7 e^	1.7 × 10^6^ ± 0.2 × 10^6 d^
IMP (100 μg/mouse)	3.2 × 10^4^ ± 1.8 × 10^4 c^	3.8 × 10^7^ ± 3.1 × 10^7 e^	2.4 × 10^8^ ± 2.3 × 10^8 f^	2.1 × 10^8^ ± 2.1 × 10^8 f^	4.7 × 10^7^ ± 1.6 × 10^7 e^
AMP (100 μg/mouse)	2.4 × 10^6^± 0.4 × 10^6 d^	5.9 × 10^7^ ± 3.6 × 10^7 e^	2.3 × 10^8^ ± 0.1 × 10^8 f^	3.0 × 10^9^ ± 1.9 × 10^9 g^	4.0 × 10^8^ ± 2.0 × 10^8 f^

### 2.4. TP3 and TP4 Do Not Exert Acute Toxic Effects in Mice

Finally, we examined the toxicity of TP3 and TP4 by delivering them via intramuscular (i.m.) injection into mice. Our results suggest that TP3 and TP4 do not induce systemic toxic effects, even at the highest concentration tested (2 mg/mouse) (Table 4). One third of mice treated with 1.5 mg or 2 mg of TP4, and one third of mice treated with 2 mg of TP3, exhibited eye narrowing (level 1 toxicity). However, one mouse injected with 2 mg of tigecycline exhibited eye narrowing, and one mouse exhibited crouching and huddling (level 2 toxicity). One of the three mice treated with 2 mg of imipenem presented with eye narrowing. Furthermore, measurement of biochemical factors in the blood revealed that intraperitoneal (i.p.) injections of low doses of TP3 or TP4 (75 μg/mice for TP3 and 25 μg/mice for TP4 (Table 5a)) did not induce any significant changes in the levels of glutamic oxaloacetic transaminase (GOT), glutamic pyruvic transaminase (GPT), blood urea nitrogen (BUN), creatinine (CRE), total bilirubin (TBIL), or uric acid (UA) (Table 5a). However, higher doses of TP3 (150 μg/mice), TP4 (50 μg/mice), or antibiotics significantly affected the levels of total bilirubin (TBIL) and uric acid (UA) (Table 5b).

**Table 4:** Gross toxicity in C57BL/6 male mice (*n* = 3 per group) treated with TP3, TP4, tigecycline (TIG), or imipenem (IMP) via intramuscular injection into the left thigh. Toxicity was graded as follows: level 1 (narrowing of eyes), level 2 (crouching and huddling), or no effect.

Dose (mg/mouse)	Mice(n) Receiving TP3	Mice(n) Receiving TP4	Mice(n) Receiving IMP	Mice(n) Receiving TIG
0.1	3, no effect	3, no effect	3, no effect	3, no effect
0.5	3, no effect	3, no effect	3, no effect	3, no effect
1	3, no effect	3, no effect	3, no effect	3, no effect
1.5	3, no effect	2, no effect; 1, toxicity level 1	3, no effect	2, no effect; 1, toxicity level 1
2	2, no effect; 1, toxicity level 1	2, no effect; 1, toxicity level 1	2, no effect; 1, toxicity level 1	1, no effect; 1, toxicity level 1; 1, toxicity level 2

## 3. Discussion

The rapid emergence and dissemination of carbapenem-resistant Enterobacteriaceae pose a considerable threat to clinical patient care and public health. Carbapenemase-producing strains are characterized by resistance to nearly all available beta-lactam antibiotics, including cephalosporins and carbapenems. Hence, these strains are frequently also resistant to other classes of antibiotics [20]. Tigecycline is more efficacious than imipenem at treating hospital-acquired pneumonia; treatment with 100 mg tigecycline resulted in more clinical cures than treatment with imipenem/cilastatin or 75 mg tigecycline [21]. Imipenem has also been reported to have induced Clostridium difficile diarrhea in a patient with chronic renal failure [22]. Although, the drug options for treating NDM-1-producing Enterobacteriaceae infections are limited, colistin and tigecycline retain some utility as agents for use against many strains, with MICs below the clinical breakpoint [23,24]. Higher doses of tigecycline (loading dose of 200 mg, then 100 mg every 24 h) have been reported to result in successful outcomes in cases of urosepsis/bacteraemia caused by MDR *K. pneumonia* or *A. baumannii*, without inducing any side effects [25]. Unfortunately, *K. pneumonia* and *A. baumannii* are becoming increasingly resistant to available antibiotics [26], demanding that we identify novel antimicrobial agents. Promising candidates include tilapia piscidin 3 (TP3) and tilapia piscidin 4 (TP4), which were isolated from *Oreochromis niloticus*, and exhibit antimicrobial activities against several bacteria [17]. In this study, we obtained *in vitro* and *in vivo* data supporting the potential of TP3 and TP4 for preventing mice infections caused by *K. pneumonia* (NDM-1) and *A. baumannii* (Sk44), as compared with conventional antibiotics.

**Table 5:** Liver and kidney functions in mice treated with the indicated compounds (control (PBS treatment)), (**a**) TP3 at 75 μg/mouse, TP4 at 25 μg/mouse; (**b**) TP3 at 150 μg/mouse, TP4 at 50 μg/mouse, tigecycline at 150 μg/mouse, imipenem at 150 μg/mouse, or ampicillin at 150 μg/mouse). The following parameters were measured in the blood: glutamic oxaloacetic transaminase (GOT), glutamic pyruvic transaminase (GPT), blood urea nitrogen (BUN), creatinine (CRE), uric acid (UA), and total bilirubin (TBIL). Each bar represents the mean value from three determinations ±standard derivation (SD). Differences were defined as significant at *p* < 0.05. Different letters indicate a significant difference between two groups, while the same letter indicates no difference between two groups.

(a)						
Treatment	GOT (U/L)	GPT (U/L)	BUN (mg/dL)	CRE (mg/dL)	UA (mg/dL)	TBIL (mg/dL)
Control	11.10 ± 4.60	6.10 ± 2.80	1.71 ± 0.09	<0.10	0.47 ± 0.06	0.11 ± 0.05
TP3	12.90 ± 5.90 (*P* > 0.05)	7.80 ± 5.00 (*P* > 0.05)	1.77 ± 0.10 (*P* > 0.05)	<0.10	0.55 ± 0.10 (*P* > 0.05)	0.19 ± 0.05 (*P* > 0.05)
TP4	7.40 ± 2.20 (*P* > 0.05)	5.80 ± 1.30 (*P* > 0.05)	1.65 ± 0.08 (*P* > 0.05)	<0.10	0.52 ± 0.09 (*P* > 0.05)	0.16 ± 0.05 (*P* > 0.05)
**(b)**						
**Treatment**	**GOT (U/L)**	**GPT (U/L)**	**BUN (mg/dL)**	**CRE (mg/dL)**	**UA (mg/dL)**	**TBIL (mg/dL)**
Control	8.67 ± 0.58 ^ab^	5.33 ± 0.58 ^ab^	1.67 ± 0.15 (*P* > 0.05)	<0.10	0.20 ± 0.1 ^a^	0.23 ± 0.06 ^c^
TP3	10.60 ± 2.51 ^a^	6.60 ± 1.52 ^b^	1.73 ± 0.11 (*P* > 0.05)	<0.10	0.50 ± 0.10 ^c^	0.20 ± 0.00 ^bc^
TP4	8.00 ± 2.00 ^ab^	6.30 ± 0.57 ^b^	1.90 ± 0.20 (*P* > 0.05)	<0.10	0.33 ± 0.05 ^abc^	0.13 ± 0.05 ^ab^
TIG	8.00 ± 1.73 ^b^	4.33 ± 0.57 ^a^	1.33 ± 1.09 (*P* > 0.05)	<0.10	0.43 ± 0.11 ^bc^	0.06 ± 0.00 ^a^
IMP	8.00 ± 1.00 ^ab^	5.30 ± 0.57 ^ab^	1.76 ± 0.15 (*P* > 0.05)	<0.10	0.26±0.11 ^ab^	0.10 ± 0.10 ^a^
AMP	8.00 ± 1.00 ^ab^	6.30 ± 0.57 ^b^	1.86 ± 0.05 (*P* > 0.05)	<0.10	0.20 ± 0.10 ^a^	0.10 ± 0.00 ^a^

We report that TP3 and TP4 are efficacious against *K. pneumonia*, *A. baumannii*, and *Pseudomonas aeruginosa* (Supplementary Figures S1–S3), and are significantly more effective than imipenem or tigecycline at treating *A. baumannii* (Sk44) or *K. pneumonia* (NDM-1) infection. This is surprising, as the MICs of TP3 against *K. pneumonia* and *A. baumannii* were <1.56–3.125 μg/mL, and the MICs of TP4 against *K. pneumonia* and *A. baumannii* were <1.56–3.125 μg/mL (MIC values could not be obtained for *K. pneumonia* (blaNDM-1)). Such MIC values would suggest that these compounds be of little use in an *in vivo* setting [27]. For example, A3-APO peptide exhibited MIC values of 8–32 μg/mL in Muller-Hinton broth against a series of clinical pathogens [28]. When administered via intramuscular injection, A3-APO (5 mg/kg) improved survival and reduced bacterial counts in the blood as compared to treatment with other antibiotics [29]. In our study, TP3 and TP4 (4× or 8× MIC) exhibited strong antimicrobial activity *in vitro*, within 60 min of exposure; the bactericidal effects of TP3 and TP4 were much stronger than those of rifampicin or human beta-defensin 3 (hBD-3). For rifampicin, bactericidal activity was achieved only at 8× MIC after 48 h of treatment [30], while hBD-3 (4 μg/mL) had bactericidal activity against *A. baumannii* after 90 min [31,32]. The differential susceptibilities of bacterial isolates to TP3 or TP4 and rifampicin or hBD-3 *in vitro* suggest that antibiotics or different AMPs may interact with the surfaces of bacterial microorganisms by different mechanisms [33]. Time-kill studies showed that TP4 has a rapid bactericidal effect against *K. pneumonia* and *A. baumannii*. This was confirmed by transmission electron microscopy (TEM) analysis, which revealed damage to bacterial cell membrane integrity, and by the measurement of 260 nm-absorbing materials in the bacterial growth media before and after peptide treatment. Taken together, these data suggest that TP4 and TP3 may induce lysis of *K. pneumonia* or *A. baumannii* cell membranes, which may prevent the development of drug resistance [34].

As multidrug resistance is commonly observed for NDM-1-producing clinical isolates, the use of AMPs acting on different targets may reduce the emergence of resistant strains and allow a reduction in therapeutic doses [35]. Although the exact mechanism of TP3 and TP4 antimicrobial function is not known, it may be related to membrane permeabilization and immunomodulation. Clinically-used antibiotics can result in the release of endotoxins, which may increase the inflammatory response [36]. On the other hand, most AMPs have immunomodulatory or LPS neutralization activity [37,38]. Therefore, TP3 and TP4 may play an important role in immunomodulatory activity against infections. Unlike ampicillin, imipenem, and tigecycline, TP3 and TP4 could protect mice against bacteremia induced by drug-resistant *K. pneumonia* (NDM-1) and *A. baumannii* (Sk44) clinical isolates. Although TP3 exhibited a loss in activity against *K. pneumonia* (NDM-1) and *A. baumannii* (Sk44) between 60 and 360 min post-injection, TP4 dramatically increased survival (60%–100%) in the bacteremia model between 30 and 180 minutes post-injection. The single doses used in this experiment (TP3 (150 μg/mouse) and TP4 (50 μg/mouse)) are compatible with concentrations used in the clinic. It is worth noting that clinically-isolated bacteria were used in our studies, which exhibit resistance to many antibiotics of general clinical use. Earlier studies have reported that administration of other AMPs after a short delay (0–120 min post-infection) increase survival [39,40]. Our results suggest that a high single dose may allow the peptide to persist for longer, thereby producing a continuous decrease in mortality. To evaluate the therapeutic potential of TP3 and TP4 for treating systemic bacterial infections, we first established a rapidly-lethal sepsis model in mice. In the infection model, TP4 significantly reduced the number of *K. pneumonia* (NDM-1) bacteria in the blood, spleen, and lymph nodes and the number of *A. baumannii* (Sk44) bacteria in the blood, peritoneum, spleen, and liver. The effects of TP4 on bacterial load were similar to those of OH-CATH peptides (10 mg/kg) on *E. coli* 25922 in infected thighs [41]. TP4 and tigecycline also had similar effects on bacterial load. However, based on the standard tigecycline daily dose regimen (100 mg loading dose plus 50 mg every 12 h), the reported target range (1.3–1.8) of *K. pneumoniae* or *E. coli* would be readily attainable at a tigecycline MIC of ≤1 μg/mL [42,43]. Comparison of our research results to the MIC distribution at 90% in the 2008 U.S. tigecycline surveillance data suggests that survival and bacteria exposure reported in our mouse model are capable of being achieved in patients infected with either organism [44]. Importantly, we do not observe acute toxic effects of TP3 or TP4 at doses of up to 2 mg/mouse, based on behavioral and biochemical analysis. However, further studies will be necessary to address adverse effects in more detail, using biomarkers to identify toxic effects on the kidneys, liver, or other organs at a greater sensitivity and for a longer period of time (e.g., daily injections of TP3 or TP4 for 30 days).

## 4. Experimental Section

### 4.1. Materials and Microorganisms

The *Pseudomonas aeruginosa* (R) strain is a clinical isolate from stool obtained from Taipei City Hospital (Heping Fuyou Branch); it is resistant to meropenem, imipenem, ciprofloxacin, cefotaxime, levofloxacin, and ampicillin/sulbactam. A second *P. aeruginosa* strain was purchased from ATCC (American Type Culture Collection, ATCC, Rockville, MD, USA; ATCC 19660). The *Acinetobacter baumannii*, *Klebsiella pneumonia*, and *Escherichia coli* strains were obtained from the Te-Li Chen (Taipei Veterans General Hospital). The features of *Acinetobacter baumannii*, *Klebsiella pneumonia*, and *Escherichia coli* strains are listed in Supplementary Table S1, and were cultured in accordance with the protocols of Dr. Te-Li Chen without modification [45,46]. All other bacterial strains were identified by routine laboratory methods and stored in 20% (v/v) glycerol at −80 °C. Mueller-Hinton broth was used as the culture medium. Reagents and chemicals were purchased from Sigma (St Louis, MO, USA). Standard laboratory powders of ampicillin (11529, USB Corporation, Cleveland, OH, USA), tigecycline (Cayman Chemical, item number 15026, USA), and imipenem (1337809; USP, Rockville, MD, USA) were used and prepared according to the guidelines of the CLSI. TP3 (H-FIHHIIGGLFSVGKHIHSLIHGH-OH), TP4 (H-FIHHIIGGLFSAGKAIHRLIRRRRR-OH), and cod Pis 1 (H-FIHHIIGWISHGVRAIHRAIHG-OH) were synthesized by solid-phase peptide synthesis, and purified by reverse-phase high-performance liquid chromatography to a grade of >95%, by GL Biochemistry (Shanghai, China). Synthetic peptides were dissolved in ddH2O for all experiments.

### 4.2. Determination of Minimum Inhibitory Concentrations (MICs) and Killing Efficiency

The MICs of TP3, TP4, and antibiotics against *Acinetobacter baumannii*, *Klebsiella pneumonia*, *Escherichia coli*, and *Pseudomonas aeruginosa* were determined using broth microdilution methods, as described previously and in accordance with NCCLS guidelines [47,48,49]. Briefly, a range of concentrations (1.56, 3.125, 6.25, 12.5, 25, 50, 100, and 200 μg/mL) of the indicated peptides and drugs were prepared by serial dilution and added to an equal volume of bacterial solution (100 μL) in each well of a 96-well plate. The plates were incubated at 37 °C for 960 min. The MIC was described as the concentration at which no microbial growth was observed visually, or via spectrophotometry at an optical density of 600 nm (U-2900, Hitachi, Japan). Negative control wells contained only growth media and microbial cells. Three biological replicates of each MIC test were performed, and each replicate was repeated three times.

To measure killing efficiency, AMP solutions with concentrations corresponding to 1×, 4×, and 8× MIC were prepared, and added to equal volumes of bacterial solution (1 × 10^5^ CFU/mL) in each well of a 96-well plate. The plates were incubated at 37 °C for 0, 30, 60, 90, 120, 150, 180, or 360 min. After the incubation period, the bacterial solutions were serially diluted in culture broth for determination of viable counts. Broth grown in the absence of peptide was used a negative control. The results are presented as mean log (CFU/mL) ± standard deviation. In vitro time-kill curve tests were performed to evaluate the bactericidal activities of TP3 and TP4 against *A. baumannii* (Sk44), *K. pneumonia* (NDM-1), *P. aeruginosa* (19660), or *P. aeruginosa* (R). Bacteria were re-suspended in fresh broth to values of l g (CFU/mL) within a range of 5–6, and the procedure was performed as previously reported [50]. Tests were carried out in triplicate, and the results are presented as mean l g (CFU/mL) ± standard derivation.

### 4.3. Assessing Bacterial Membrane Integrity in the Presence of TP3 and TP4

The membrane integrities of *Acinetobacter baumannii* and *Klebsiella pneumonia* were determined by measuring the release of nucleic acids from ruptured cells [50,51]. Equal volumes (0.5 mL) of bacterial suspension (3 × 10^8^ CFU/mL) and peptide solution (at 1 ×, 2 ×, or 4 × MIC) were mixed and incubated for 60 min. PBS solution was used as a negative control. After incubation, suspensions were poured through a 0.22 μm filter to isolate the bacteria from the supernatant; the absorbance of the suspension at 260 nm was subsequently measured. Three biological replicates were performed for each experiment, and triplicate measurements were made for each condition. The experimental data were normalized against the absorbance of supernatant of untreated cells in PBS at the same wavelength. Bacterial membrane integrity was examined by transmission electron microscopy (TEM). *Acinetobacter baumannii* and *Klebsiella pneumonia* were either untreated or treated with TP4 (100 μg/mL) for 60 min, and samples were then analyzed and prepared for TEM in accordance with a published report [47].

### 4.4. Mouse Model of Sepsis

Male C57BL/6 mice (21.89 ± 1.33 g) were provided by the LASCO company (Taipei, Taiwan). All procedures, care, and handling of mice were approved by the laboratory animal ethics committee of Southern Taiwan University. To induce sepsis, mice were challenged with *Acinetobacter baumannii* (Sk44; 3 × 10^7^ CFU/mL, 100 µL/mouse) or an NDM-1-producing strain of *Klebsiella pneumonia* (NDM-1; 1.5 × 10^8^ CFU/mL, 100 µL/mouse) by intraperitoneal (ip) injection. The bacterial strains are listed in Supplementary Table S1. At 30 min after injection of bacteria, mice were treated with ampicillin (150 μg/mouse), imipenem (150 μg/mouse), tigecycline (150 μg/mouse), TP3 (150 μg/mouse), or TP4 (50 μg/mouse) by ip injection; survival rate was monitored for up to 168 h. Each experimental group consisted of 6–10 mice, and three biological replicates were performed for each condition.

Time-dependent effects were examined by treating mice (*n* = 5) with TP3 (150 μg/mouse) or TP4 (50 μg/mouse) via ip injection at 30, 60, 180, or 360 min after injection with *Acinetobacter baumannii* (Sk44; 3 × 10^7^ CFU/mL) or NDM-1-producing Klebsiella pneumonia (NDM-1; 1.5 × 10^8^ CFU/mL). Survival rate and mouse status were monitored from 24–168 h. To examine bacterial dissemination and killing rate, mice (*n* = 5) were infected with *Acinetobacter baumannii* (Sk44; 1 × 10^7^ CFU/mL, 100 μL/mouse) or an NDM-1-producing strain of *Klebsiella pneumonia* (NDM-1; 1 × 10^7^ CFU/mL, 100 μL/mouse); at 30 min after infection, mice were treated with antibiotics, TP3, or TP4 for a further 72 h. After the incubation period, mice were sacrificed for examination of bacterial dissemination and killing rate Control mice (*n* = 5) were injected with bacteria, but untreated. Bacterial numbers in blood, peritoneum, spleen, liver, and mesenteric lymph nodes were recorded. Colony counts from the diluted bacterial solutions were expressed relative to those at the start of treatment.

### 4.5. In Vivo Toxicity

To determine the toxicity of TP3 or TP4, TP3 and TP4 were dissolved in ddH2O, and administered as intramuscular bolus injections in the left thigh (0.1, 0.5, 1, 1.5, or 2 mg/mouse). Mice were observed for signs of systemic toxicity.

To study the effect of treatment on biochemistry, mice (*n* = 10 in each group) were treated with PBS (control), TP3, TP4, imipenem (IMP), ampicillin, or tigecycline (TIG) twice daily for 3 days. TP3, TP4, IMP, ampicillin, or TIG were delivered via ip injection (100 μL of the indicated solution/mouse). TP3 was delivered at 75 or 150 μg/mouse, TP4 was delivered at 25 or 50 μg/mouse, ampicillin at 150 μg/mouse, TIG at 150 μg/mouse, and IMP at 150 μg/mouse. TP3 and TP3 were dissolved in ddH2O, while the antibiotics were dissolved in physiological salt solution. Blood samples (0.2 mL) were collected 24 h after the final injection of TP3, TP4, TIG, IMP, or AMP, and used to determine the serum levels of glutamic oxaloacetic transaminase (GOT), glutamic pyruvic transaminase (GPT), blood urea nitrogen (BUN), creatinine (CRE), total bilirubin (TBIL), and uric acid (UA).

## 5. Conclusions

The data presented here indicate that TP3 and TP4 significantly increase the survival rate of mice infected with *A. baumannii* (Sk44) or *K. pneumonia* (NDM-1) as compared with untreated controls or mice treated with antibiotics. Furthermore, these AMPs reduced bacterial burden in the blood and other organs within 72 h. Critically, TP3 and TP4 did not affect biochemical parameters at ED50 or ED100. Thus, TP3 and TP4 are promising compounds for treating bacterial infections caused by carbapenem-resistant *A. baumannii* (Sk44) or NDM-1-producing *K. pneumonia*, pending further studies.

## Supplementary Files

Supplementary File 1
