# Supplementary material for: Piscidin is Highly Active against Carbapenem-Resistant Acinetobacter baumannii and NDM-1-Producing Klebsiella pneumonia in a Systemic Septicaemia Infection Mouse Model
Source: Mar Drugs. 2015 Apr 14;13(4):2287–305. doi: 10.3390/md13042287 (PMC4413212; doi:10.3390/md13042287)
Supplement: Supplementary File 1 [file marinedrugs-13-02287-s001.pdf]

Supplementary Information

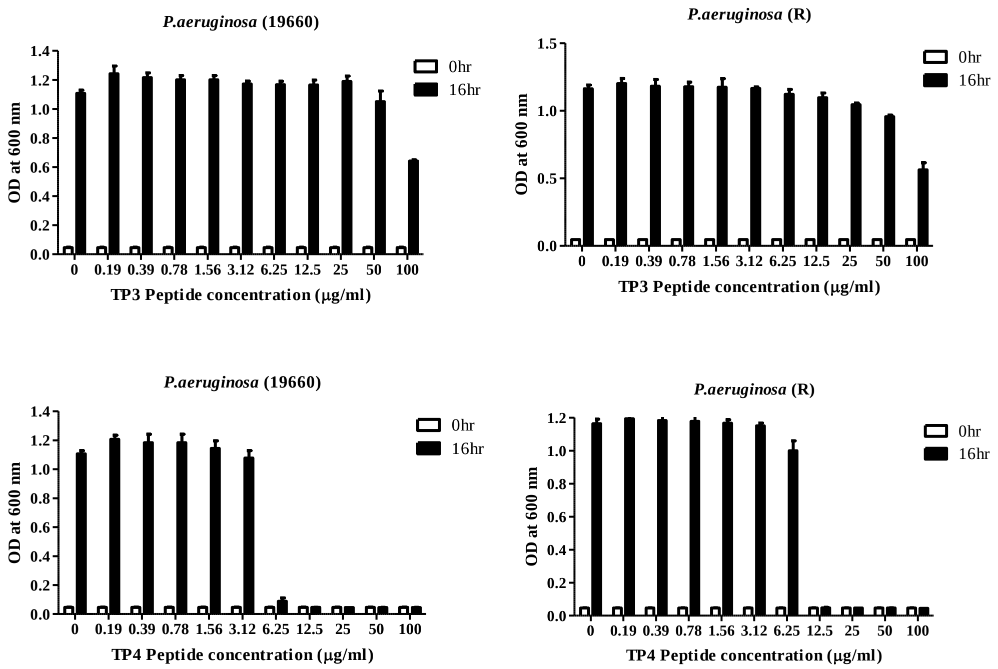

Figure S1. Dose-dependent growth inhibition of Pseudomonas aeruginosa (ATCC19660) or drug-resistant Pseudomonas aeruginosa (R) by incubation with TP3 or TP4 for 16 h. The data are expressed as means of three replicates. Error bars represent the standard derivation (SD). OD, optical density.

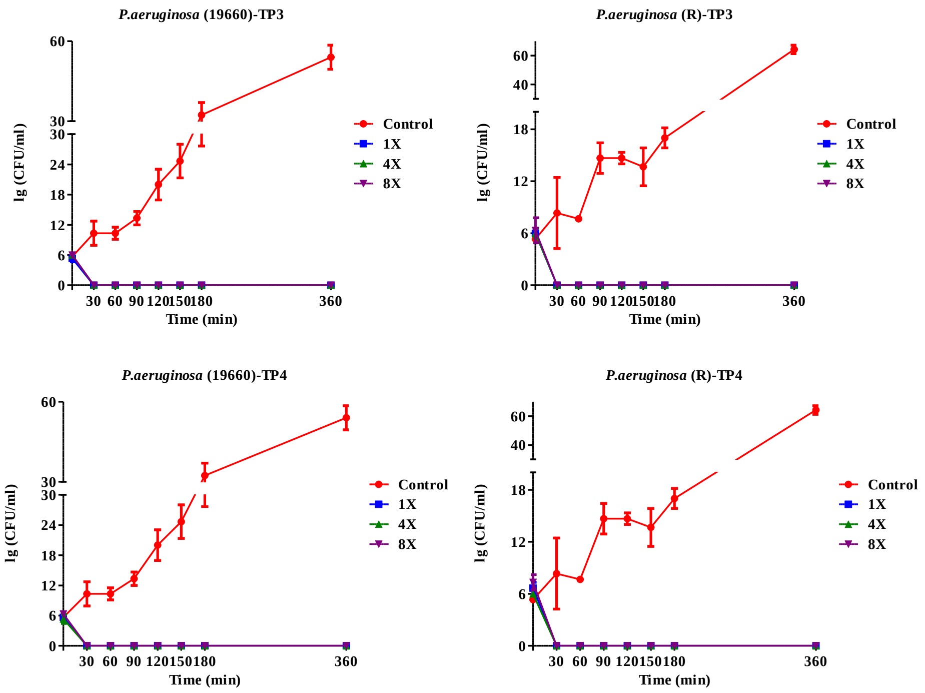

Figure S2. Kill kinetics of TP3 and TP4 against Pseudomonas aeruginosa (ATCC19660) and drug-resistant Pseudomonas aeruginosa (R). Controls were not treated with peptide. The peptide concentrations were 1 × MIC (1 ×), 4 × MIC (4 ×), or 8 × MIC (8 ×). Experiments were performed in triplicate and the bactericidal activities are presented as mean lg (CFU/mL). Error bars represent the standard deviations.

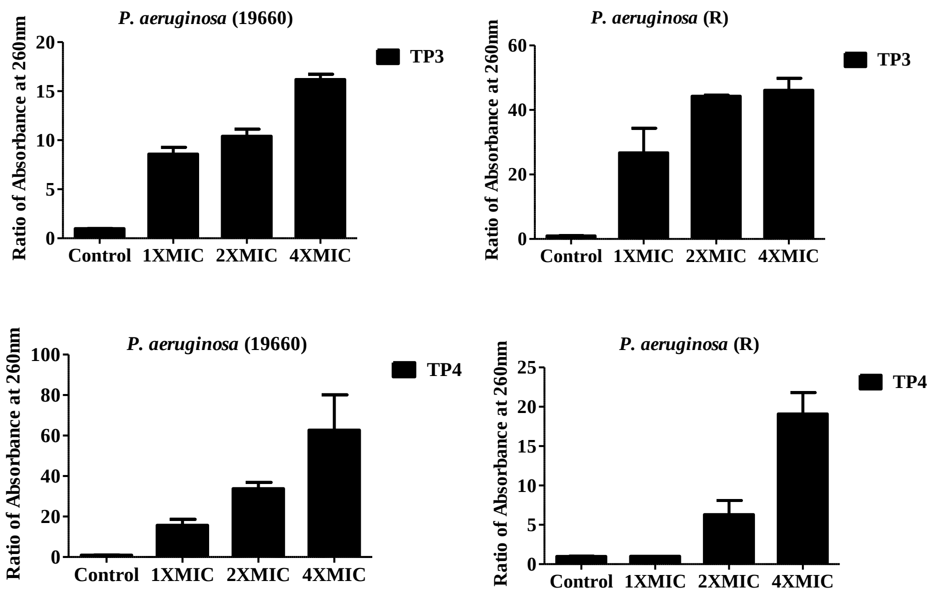

Figure S3. The effect of TP3 and TP4 on the release of DNA from Pseudomonas aeruginosa (ATCC19660) or drug-resistant Pseudomonas aeruginosa (R). Peptide concentrations used were 1 × MIC, 2 × MIC, or 4 × MIC. “Control” indicates untreated cells. The data are means of at least three independent experiments. Error bars represent the standard deviations.

Table S1. Phenotypes, genotypes, and drug resistant features of clinical bacteria isolates provided by Te-Li Chen (Taipei Veterans General Hospital) for use in this study.

Bacterial strain	Information
K. pneumoniae (YT 32)	Phenotype: blaAmpC (+)<br>Genotype: blaAmpC (+) ESBL (+)
E. coli (YT 39)	Phenotype: blaAmpC (+)<br>Genotype: blaAmpC (+)
E. coli (YT 154)	Phenotype: blaAmpC (+)<br>Genotype: blaAmpC (+) ESBL (+)

© 2015 by the authors; licensee MDPI, Basel, Switzerland. This article is an open access article distributed under the terms and conditions of the Creative Commons Attribution license (http://creativecommons.org/licenses/by/4.0/).
